# The effect of infrared beak treatment on the welfare of turkeys reared to 12 weeks of age

**DOI:** 10.1016/j.psj.2022.101728

**Published:** 2022-01-13

**Authors:** S. Struthers, T. Fiss, H.L. Classen, S. Gomis, R. Dickinson, T.G. Crowe, E. Herwig, K. Schwean-Lardner

**Affiliations:** ⁎ Roslin Institute, Royal (Dick) School of Veterinary Studies, University of Edinburgh, Edinburgh EH25 9RG, Scotland; † Monogastric Science Research Centre, Scotland's Rural College, Roslin Institute, EH25 9RG Scotland; ‡ Department and Animal and Poultry Science, University of Saskatchewan, Saskatoon, Canada S7N 5A8; § Department of Veterinary Pathology, Western College of Veterinary Medicine, University of Saskatchewan, Saskatoon, Canada S7N 5B4; # Department of Mechanical Engineering, University of Saskatchewan, Saskatoon, Canada S7N 5A9

**Keywords:** beak, behavior, histology, turkey poult, beak shape

## Abstract

This study aimed to determine the effects of infrared beak treatment on the behavior and welfare of male and female turkeys reared to 12 wk of age. To do this, poults (236 males and 324 females) were assigned to one of 2 beak treatments: infrared beak treated on day of hatch (**IR**) or sham untreated control (**C**). Data collected included heterophil/lymphocyte (**H/L**) ratio, pecking force, feather cover, behavioral expression, and beak histology. Data were analyzed as a 2 × 2 factorial of beak treatment and gender, in a completely randomized design and analyzed using PROC MIXED (SAS 9.4). H/L ratio (indicative of a stress response) did not differ between treated and control poults during early life, except at 20 d of age when H/L ratio was higher for C poults than IR poults. Pecking force, measured as a method of monitoring pain, was different only at 1 wk of age, when IR poults pecked with more force than C poults. Feather cover was better in IR poults at 12 wk of age. Differences in behavior between treatments were minor over the 12-wk period. Overall, infrared beak treatment of commercial turkeys had minimal negative impacts on behavior and welfare. The results suggest that stress may be reduced in flocks that are beak treated and that the procedure itself does not cause a pain response.

## INTRODUCTION

Beak treatment is a common management practice used to control cannibalism and injurious pecking behavior in commercial layers, broiler breeders, and turkeys (Jendral and Robinson, 2004). In the past, beak treatment was commonly performed using a hot blade which simultaneously trimmed and cauterized the beak tissue (Jendral and Robinson, 2004). Hot-blade trimming resulted in acute pain and depending on the age and the severity of the trim, may have resulted in chronic pain (Lunam et al., 1996). Infrared beak treatment is a newer technology that uses an infrared light to treat the beak tissue without creating an open wound. The infrared light penetrates the outer layer of the beak, damaging the tissue layers underneath and inhibiting further growth of the beak tip (Dennis et al., 2009). The loss of the beak tissue is gradual as the beak tip sloughs off over a period of approximately 1 to 3 wk post-treatment (Struthers et al., 2019b). This allows birds to adapt to the change in beak shape and use their beaks normally during the critical first few days of life.

Although infrared beak treatment has been reported to be more welfare-friendly alternative to hot-blade trimming (Dennis et al., 2009), societal concern still exists regarding any form of beak modification. Some of the major concerns are that the practice may cause chronic pain and stress, impaired function of the beak, and a reduced ability to perform behaviors such as feeding, drinking, and preening (Kuenzel, 2007). Heterophil/lymphocyte (**H/L**) ratio is considered to be a reliable indicator of chronic stress (Gross and Siegel, 1983). In laying hens, leaving hens with untreated, intact beaks resulted in higher H/L ratios compared to hens that were hot-blade trimmed at 1 d, 10 d, or 10 wk of age (Onbasilar et al., 2009). Dennis et al. (2009) reported that infrared beak treatment did not negatively affect H/L ratio and immune function when compared to hot-blade trimming. If birds are in pain post-beak treatment, they may use less force when pecking at food or novel objects. Both Freire et al. (2008) and Struthers et al. (2019b) found that infrared beak-treated pullets did not show reductions in pecking force throughout rearing, suggesting that infrared beak treatment did not cause a pain response in the beak. To the author's knowledge, H/L ratio and pecking force in relation to beak treatment has not been studied in turkeys.

Beak treated birds may be less successful at pulling and removing the feathers of others, which reduces the risk of cannibalism (Riber and Hinrichsen, 2017; Struthers et al., 2019b). It has been found in both commercial turkeys and laying hens, that beak treated birds have better feather cover compared to birds with intact beaks (Denbow et al., 1984; Leighton et al., 1985; Onbasilar et al., 2009; Morrissey et al., 2016; Riber and Hinrichsen, 2017; Struthers et al., 2019b). Regardless of method, beak treatment appears to have short-term effects on behavior. Male and female turkeys that were electrically beak trimmed at one-day of age showed reduced feeding and drinking and increased inactivity for up to 2 wk post-treatment (Cunningham et al., 1992). This has also been observed in studies of infrared beak-treated layer pullets (Marchant-Forde et al., 2008); however, more recently, Struthers et al. (2019b) found that infrared beak-treated layer pullets were more active compared to untreated pullets during the first 4 wk post-treatment.

Considerable research has been published on the effect of infrared beak treatment on laying hens, however, there is a lack of equivalent research published for turkeys. Therefore, the objective of this study was to determine the impact of infrared beak treatment and gender on the behavior and welfare of turkeys raised to 12 wk of age. To the author's knowledge, this is the first study examining infrared beak treatment in turkeys and its effect on welfare.

## MATERIALS AND METHODS

This work was approved by the University of Saskatchewan's Animal Research Ethics Board and adhered to the Canadian Council on Animal Care guidelines for humane animal use (2009). The present study was part of a larger study examining the effects of infrared beak treatment on the productivity and welfare of turkeys raised to 12 wk of age. While the present study's focus is primarily on behavior and welfare, the productivity data manuscript is currently under review (Struthers et al.).

### Birds and Housing

Nicholas Select turkeys (n = 506; 236 males and 324 females) were hatched and sexed at a commercial hatchery. Poults were randomly assigned to one of 2 beak treatments: infrared beak treated (**IR**) or sham untreated control (**C**). Poults were beak treated using the Poultry Service Processor (Nova-Tech Engineering LLC, Willmar, MN) at a lamp power of 35 and no reflective mirror, resulting in only the top beak being treated. Poults in the C treatment were handled and loaded on the infrared beak treatment equipment to simulate conditions experienced by the treated poults; however, their beak tips were not exposed to the infrared light. Upon arrival at the research facility, poults were housed in floor pens (n = 16; 3 m × 3 m) and a brooder ring (8 cm circumference) was used for the first 10 d. Stocking density for the pens was based on the predicted 12-wk body weight and was a maximum of 32 kg/m^2^ (27 male per pen and 38 females per pen) (Aviagen, 2015). The remaining 10 birds per treatment were placed into 2 separate pens based on beak treatment and were later euthanized to collect beak samples for histological analyses.

Supplemental feeders and drinkers were provided for each pen for the first week. Each pen also had a heat lamp for the first two weeks. Wood shavings were used as bedding and wheat straw was provided equally to all pens after brooding if litter quality was poor. Poults were given ad libitum access to age-appropriate commercial diets in tube feeders (1 per pen; 36 cm diameter for first 4 wk and 44 cm thereafter). Ad libitum water access was provided using bell drinkers (1 per pen; 38 cm diameter for first 4 wk and 56 cm thereafter). For the initial brooding period, temperature was 30°C. Temperature was reduced by approximately 2°C per week to reach a final temperature of 13°C at 12 wk of age. Photoperiod and light intensity were 23L:1D (40 lux) for the first 5 d and then reduced by one hour of light and 6 lux per day to reach a final photoperiod of 18L:6D (5 lux) at 10 d of age.

### Data Collection

Blood samples were collected for H/L ratio determination from 10 turkeys per treatment from the jugular vein at 1, 5, and 10 d of age, and the brachial vein at 15 and 20 d of age. Blood smears were prepared on the same day blood was collected. The slides were allowed to dry and then stained with PROTOCOL Hema 3 (Fisher Scientific, Ottawa, ON, Canada). Slides were analyzed under 100× oil magnification and all heterophils and lymphocytes within a field of view were counted until a total of 100 cells was counted (microscope B-290TB, Optika, Bergamo, Italy).

The force that poults used to peck at food objects was measured weekly for the first 4 wk of the experimental period. Six turkeys per treatment were randomly chosen and removed from their home pen. Birds were weighed to determine BW and then placed into an enclosure (40 cm × 40 cm) within the same room as their home pen, that contained a force plate connected to a load cell, which was connected to a P-3500 Portable Strain Indicator unit (Vishay Measurements Group, Raleigh, NC) and visualized on a TDS1002R oscilloscope (Tektronix Inc., Beaverton, OR) situated in the center. Three pecks were recorded and averaged per bird where a peck was considered any hit from the beak onto the force plate (load cell). Once a bird had pecked the force plate, the force (measured in millivolts [mV]) was recorded and converted to newtons (**N**) by multiplying the mV value by the sensitivity reciprocal (see below).

$$\text{Sensitivity}\;\text{reciprocal}=\frac{(m/1000)*\;9.81}{\text{System}\;\text{output}}=\;\frac{(1000/1000)\;*9.81}{640}=\;0.01532815$$

$$\begin{array}{c} m=\text{known}\;\text{mass}=1000\;g\\ \text{System}\;\text{output}=640\;\text{mV} \end{array}$$

A total of 54 males and 76 females per treatment were individually scored for feather cover at 8 and 12 wk of age by the same 2 independently working trained evaluators (both scoring each bird). Five body areas were scored: neck, back, breast, wings, and tail. These areas were given a score of 1 (0–25% feather cover), 2 (26–50% feather cover), 3 (51–75% feather cover), or 4 (75%-full plumage) using a scale adapted from Davami et al. (1987). Feather cover scores were calculated as an average of the scores given by each independent scorer for statistical analyses.

Behavior on d 1, 6, and 8 of age was recorded for 24 continuous hours from 2 replicates per treatment using HFR700 camcorders (Canon Canada, Mississauga, ON, Canada). These camcorders were unable to capture the entire pen, so any bird not present on the screen was recorded as unknown. At 3, 8, and 12 wk of age, behavior from 2 replicates per treatment was recorded for 24 continuous hours using ceiling mounted infrared video camera systems (Matrix Network Inc., Coppell, TX) that captured the whole pen. Videos were analyzed using scan sampling at 15-min intervals. Behavioral expression was classified using the ethogram described in Table 1.

**Table 1:** Ethogram of behaviors commonly performed by commercial turkeys.

Behavior	Description^1^
Resting	Lying down, otherwise inactive; eyes may be open or closed
At the feeder	Head extended into the feeder; manipulating or ingesting feed
At the drinker	Head extended into the drinker
Dustbathing	The turkey lies on its breast and shows rapid wing movements, body shaking, and/or rhythmic leg movements
Aggressive pecking	Pecks delivered to the head or body that cause the receiving bird to move away
Gentle pecking	Pecks directed toward other birds that does not cause harm or damage
Vent pecking	Pecks directed toward the vent region of other birds; can result in tissue damage and cannibalism
Cannibalism	Pecking at, tearing, and consumption of blood and body tissues of conspecifics
Preening^2^	Grooming own feathers with beak while standing or laying
Wing flapping^2^	Extension of wings away from body and flapping up and down rapidly but without flight/walking
Stretching^2^	Extension of wings away from body without flapping or walking
Foraging^3^	Scratching and pecking at the litter
Environment pecking^3^	Pecking at objects in the environment (feeder, drinker, litter, walls)
Stuck upside-down (flip)^4^	The turkey is on its back unable to right itself
Head shaking^4^	Head is moved side to side/up and down rapidly
Beak rubbing^4^	Rapid stroking of alternate sides of the beak
Strutting^4^	Erecting the back feathers and walking with the wings held to the side
Standing^5^	Standing and idle; eyes may be open or closed
Walking^5^	Taking at least 2 successive steps
Unknown^4^	Behavior cannot be discerned because bird is not visible or is being blocked by other birds

1 Adapted from van Liere and Wiepkema (1992), Martrenchar et al., (2001), Cloutier et al., (2002), and Marchant-Forde et al. (2008).

2 Denotes a comfort behavior.

3 Denotes an exploratory behavior.

4 Denotes a low incidence behavior.

5 Denotes an active behavior.

On 1, 5, 10, 15, and 20 d of age, beak samples from 2 birds per treatment were collected. Birds were humanely euthanized by manual cervical dislocation and their beaks were removed by cutting where the beak attached to the skull. Beaks were then placed in 10% neutral buffered formalin and stored at room temperature. Beaks were trimmed into sagittal cross sections and placed in cassettes. Samples were submitted to an independent diagnostic laboratory for slide preparation (embedded in paraffin wax, sectioned at 5 µm, and stained with hematoxylin and eosin (H&E; SelecTech Hematoxylin 560 and SelecTech Alcoholic Eosin Y515, Leica Biosystems, Winnipeg, MB, Canada). Beaks from d 15 and 20 were decalcified in 20% formic acid for 24 h prior to sectioning.

### Statistical Analyses

The experimental design for this study was a 2 × 2 factorial arrangement of beak treatment and gender, in a completely randomized design with 4 pen replicates per treatment. The experimental unit used for feather cover and behavior was pen. The experimental unit used for H/L ratio and pecking force was bird. Data were analyzed with ANOVA using PROC MIXED (SAS9.4, Cary, NC) with Tukey's range test to separate means. Percentage data was checked for normality using PROC UNIVARIATE (SAS 9.4) and log transformed (data log + 1) when necessary. Differences were considered significant when *P* ≤ 0.05 and a trend was noted when 0.05 < *P* ≤ 0.10.

## RESULTS

### Heterophil/Lymphocyte Ratio

Leaving turkeys with intact beaks resulted in a higher H/L ratio at 20 d of age (Table 2). No differences in H/L ratio were found between males and females. On d 15, there was an interaction between beak treatment and gender for H/L ratio with IR males having lower ratios than C males (0.31 vs. 0.40, respectively) and C females have lower ratios than IR females (0.32 vs. 0.39, respectively).

**Table 2:** Effect of infrared beak treatment and gender on the heterophil/lymphocyte ratio of turkeys at 1, 5, 10, 15, and 20 d of age.

Age (d)	Beak treatment			Gender			Interaction	SEM
	IR	C	*P*-value	Male	Female	*P*-value	*P*-value	
1	0.39	0.46	0.33	0.47	0.39	0.30	0.32	0.038
5	0.36	0.46	0.12	0.43	0.39	0.47	0.07	0.032
10	0.52	0.41	0.21	0.52	0.41	0.22	0.93	0.036
15	0.35	0.36	0.77	0.35	0.35	0.93	0.04	0.023
20	0.58^b^	0.76^a^	0.01	0.71	0.63	0.27	0.86	0.035

IR, infrared beak treated.

C, sham untreated control.

a,b Means within a main effect with different superscripts are significantly different (*P* ≤ 0.05).

### Pecking Force

Infrared beak treatment had minimal impact on the force with which turkeys used to peck at food objects (Table 3). At one week of age, IR turkeys pecked with more force compared to C poults (14 vs. 6 N, respectively) but did not differ from C poults after this age. No differences in pecking force were found between males and females throughout the testing period.

**Table 3:** Effect of infrared beak treatment and gender on the pecking force (N) of turkeys from 1 to 4 weeks of age.

Age (wk)	Beak treatment			Gender			Interaction	SEM
	IR	C	*P*-value	Male	Female	*P*-value	*P*-value	
1	14^a^	6^b^	<0.01	10	11	0.68	0.14	1.5
2	18	18	0.86	19	16	0.11	0.06	1.0
3	25	24	0.76	26	23	0.32	0.95	1.3
4	32	31	0.59	32	31	0.69	0.75	1.7

IR, infrared beak treated.

C, sham untreated control.

a,b Means within a main effect with different superscripts are significantly different (*P* ≤ 0.05).

### Feather Cover

At 8 wk of age, feather cover on the back was better in IR turkeys compared to C (scores of 3.0 vs. 2.9, respectively) while feather cover of the wings was better in C than IR turkeys (3.7 vs. 3.5, respectively; Table 4). Interactions between beak treatment and gender were noted for neck and overall feather cover scores this age. Male IR turkeys had poorer neck feather cover compared to C males and C females, and poorer overall feather cover compared to C males (Table 5). At 12 wk of age, IR turkeys had better feather cover on the breast and tail compared to C (Table 4). Interactions were noted for the neck, back, wings, and overall feather cover scores. Males, regardless of treatment, and IR females had better feather cover on the neck compared to C females (Table 5). IR females had better feather cover on the back compared to IR males and C females, better wing feather cover compared to IR males, and better overall feather cover compared to all other treatments.

**Table 4:** Effect of infrared beak treatment and gender on the average feather cover score (scale 1–4)^1^ of turkey toms and hens at 8 and 12 wk of age.

Age (wk)	Beak treatment			Gender			Interaction	SEM
	IR	C	*P*-value	Male	Female	*P*-value	*P*-value	
8 wk of age								
Neck	3.4^b^	3.7^a^	<0.01	3.5	3.5	0.72	0.02	0.03
Back	3.0^a^	2.9^b^	<0.01	3.0	2.9	0.08	0.50	0.02
Breast	4.0	4.0	0.40	4.0	4.0	0.33	0.40	0.01
Wings	3.5^a^	3.7^a^	0.03	3.6	3.6	0.42	0.18	0.03
Tail	3.0	3.0	0.88	3.0	3.0	0.97	0.05	0.02
Overall^2^	16.9^b^	17.2^a^	<0.01	17.1	17.1	0.74	<0.01	0.06
12 wk of age								
Neck	3.9^a^	3.8^b^	0.01	3.9^a^	3.8^b^	0.01	0.02	0.02
Back	2.3	2.2	0.38	2.2	2.3	0.11	<0.01	0.02
Breast	4.0^a^	3.9^b^	0.03	4.0	4.0	0.42	0.19	0.01
Wings	3.3	3.3	0.27	3.3	3.3	0.10	0.03	0.02
Tail	3.2^a^	3.0^b^	0.01	3.0^b^	3.2^a^	0.02	0.97	0.03
Overall^2^	16.6^a^	16.3^b^	0.01	16.4	16.5	0.18	<0.01	0.05

IR, infrared beak treated.

C, sham untreated control.

a,b Means within a main effect with different superscripts are significantly different (*P* ≤ 0.05).

1 Score of 1 = 0–25% feather cover, 2 = 26–50% feather cover, 3 = 51–75% feather cover, and 4 = 76%-full, intact plumage (Davami et al., 1987).

2 Sum of 5 body areas (total out of 20): neck, back, breast, wings, and tail.

**Table 5:** Interaction between infrared beak treatment and gender on the feather cover score of turkey toms and hens at 8 and 12 wk of age.

Body area	Male IR	Male C	Female IR	Female C
8 wk of age				
Neck	3.3^c^	3.8^a^	3.4^bc^	3.6^ab^
Overall	16.8^b^	17.5^a^	17.1^ab^	17.1^ab^
12 wk of age				
Neck	3.9^a^	3.9^a^	3.9^a^	3.7^b^
Back	2.2^b^	2.3^ab^	2.4^a^	2.2^b^
Wings	3.2^b^	3.3^ab^	3.4^a^	3.3^ab^
Overall	16.4^b^	16.4^b^	16.8^a^	16.2^b^

IR, infrared beak treated.

C, sham untreated control.

a-c Means within a row with different superscripts are significantly different (*P* ≤ 0.05).

### Behavior

There was minimal impact of infrared beak treatment on turkey behavior during early life. At 1-d of age, IR turkeys spent more time resting but less time performing low incidence behaviors compared to C turkeys (Table 6). At 6 d of age, IR turkeys spent less time exploring the environment compared to C turkeys (0.94 vs. 1.89 percent of time, respectively). IR turkeys spent more time gentle pecking compared to C turkeys at 8 d of age (0.07 vs. 0.02 percent of time, respectively). Later in life, the only effect of infrared beak treatment on behavior was at 3 wk of age with IR turkeys spending more time at the feeder compared to C (7.26 vs. 5.53 percent of time, respectively).

**Table 6:** Effect of infrared beak treatment and gender on the behavior (percent of time) of turkey toms and hens.

Behavior	Beak treatment			Gender			Interaction	SEM
	IR	C	*P*-value	Male	Female	*P*-value	*P*-value	
1 d of age								
Resting	62.34^a^	54.29^b^	<0.01	56.55^b^	60.08^a^	0.03	0.15	1.811
Feeding	5.60	7.44	0.23	6.92	6.11	0.80	0.97	0.619
Drinking	0.76	0.73	0.81	0.62	0.87	0.16	0.59	0.106
Dustbathing	0.00	0.05	0.37	0.05	0.00	0.37	0.37	0.024
Aggressive pecking	0.04	0.06	0.61	0.05	0.06	0.56	0.80	0.013
Gentle pecking	0.06	0.04	0.94	0.07	0.03	0.79	0.81	0.022
Vent pecking	0.00	0.00	-	0.00	0.00	-	-	0.000
Cannibalism	0.00	0.00	-	0.00	0.00	-	-	0.000
Comfort	0.50	0.49	0.77	0.57	0.41	0.61	0.23	0.071
Exploratory	0.39	0.24	0.70	0.29	0.34	0.77	0.74	0.114
Low incidence	11.97^b^	18.78^a^	0.02	16.13	14.61	0.70	0.28	1.650
Active	18.35	17.88	0.81	18.74	17.48	0.89	0.48	0.671
6 d of age								
Resting	34.52	40.66	0.38	41.43	33.76	0.26	0.79	2.736
Feeding	3.77	4.44	0.51	4.46	3.75	0.72	0.10	0.380
Drinking	1.23	1.43	0.67	1.22	1.43	0.41	0.90	0.160
Dustbathing	0.00	0.01	0.37	0.01	0.00	0.37	0.37	0.005
Aggressive pecking	0.04	0.09	0.40	0.06	0.07	0.69	0.82	0.022
Gentle pecking	0.02	0.07	0.29	0.05	0.03	0.87	0.92	0.019
Vent pecking	0.00	0.00	-	0.00	0.00	-	-	0.000
Cannibalism	0.00	0.00	0.37	0.00	0.00	0.37	0.37	0.002
Comfort	1.18	1.32	0.10	1.17^b^	1.34^a^	0.01	0.05	0.068
Exploratory	0.94^b^	1.89^a^	0.04	1.56	1.27	0.71	0.72	0.208
Low incidence	29.04	21.67	0.13	22.00	28.71	0.15	0.70	2.532
Active	29.27	28.41	0.26	28.05	29.63	0.11	0.20	0.924
8 d of age								
Resting	37.58	38.89	0.81	41.64	35.84	0.34	0.47	1.771
Feeding	3.40	4.39	0.34	4.33	3.69	0.99	0.61	0.296
Drinking	1.21	0.97	0.19	1.32	0.88	0.32	0.85	0.128
Dustbathing	0.01	0.00	0.49	0.00	0.01	0.49	0.49	0.004
Aggressive pecking	0.05	0.01	0.38	0.00	0.04	0.20	0.38	0.015
Gentle pecking	0.07^a^	0.02^b^	0.05	0.05	0.03	0.17	0.20	0.015
Vent pecking	0.00	0.00	-	0.00	0.00	-	-	0.000
Cannibalism	0.00	0.00	-	0.00	0.00	-	-	0.000
Comfort	1.20	1.14	0.98	0.95^b^	1.32^a^	0.03	0.54	0.098
Exploratory	0.90	1.30	0.18	1.44	0.89	0.15	0.80	0.161
Low incidence	19.24	20.78	0.54	17.72	21.92	0.20	0.75	1.568
Active	36.35	32.52	0.13	32.54	35.38	0.13	0.70	1.073
3 wk of age								
Resting	57.88	59.70	0.37	60.37	57.21	0.07	0.49	1.001
Feeding	7.26^a^	5.53^b^	0.01	6.32	6.47	0.06	0.99	0.305
Drinking	0.92	0.96	0.17	0.96	0.91	0.28	0.31	0.042
Dustbathing	0.01	0.01	0.98	0.01	0.01	0.50	0.98	0.004
Aggressive pecking	0.00	0.03	0.28	0.04	0.00	0.12	0.28	0.013
Gentle pecking	0.00	0.00	-	0.00	0.00	-	-	0.000
Vent pecking	0.00	0.00	-	0.00	0.00	-	-	0.000
Cannibalism	0.00	0.00	-	0.00	0.00	-	-	0.000
Comfort	0.06	0.04	0.49	0.04	0.06	0.38	0.95	0.011
Exploratory	0.05	0.04	0.89	0.02	0.06	0.25	0.41	0.022
Low incidence	2.57	3.14	0.50	2.84	2.86	0.86	0.63	0.312
Active	31.25	30.55	0.99	29.40	32.40	0.15	0.85	0.905
8 wk of age								
Resting	65.11	66.24	0.87	69.36^a^	61.99^b^	0.01	0.68	1.579
Feeding	2.96	2.66	0.49	2.20^b^	3.42^a^	<0.01	0.55	0.205
Drinking	1.19	1.92	0.28	1.76	1.36	0.94	0.33	0.309
Dustbathing	0.00	0.01	0.18	0.01	0.01	0.91	0.91	0.004
Aggressive pecking	0.02	0.01	0.62	0.02	0.01	0.63	0.34	0.007
Gentle pecking	0.03	0.01	0.30	0.01	0.03	0.24	0.79	0.009
Vent pecking	0.00	0.00	-	0.00	0.00	-	-	0.000
Cannibalism	0.00	0.00	-	0.00	0.00	-	-	0.000
Comfort	0.30	0.18	0.29	0.26	0.22	0.85	0.17	0.054
Exploratory	0.10	0.05	0.22	0.12	0.03	0.11	0.06	0.028
Low incidence	0.66	0.75	0.54	0.41^b^	0.99^a^	<0.01	0.30	0.096
Active	29.62	28.18	0.49	25.85^b^	31.95^a^	0.04	0.29	1.450
12 wk of age								
Resting	70.87	69.99	0.52	74.90^a^	65.96^b^	<0.01	0.99	1.343
Feeding	3.22	3.26	0.34	3.19	3.29	0.07	0.19	0.134
Drinking	1.24	1.41	0.45	1.35	1.31	0.42	0.40	0.096
Dustbathing	0.00	0.01	0.22	0.01	0.00	0.58	0.58	0.006
Aggressive pecking	0.05	0.01	0.12	0.04	0.01	0.23	0.27	0.012
Gentle pecking	0.04	0.02	0.31	0.03	0.03	0.90	0.64	0.012
Vent pecking	0.00	0.00	-	0.00	0.00	-	-	0.000
Cannibalism	0.00	0.00	-	0.00	0.00	-	-	0.000
Comfort	0.43	0.52	0.74	0.67	0.28	0.32	0.93	0.139
Exploratory	0.04	0.04	0.88	0.06	0.02	0.14	0.88	0.013
Low incidence	0.46	0.65	0.25	0.21^b^	0.90^a^	<0.01	0.18	0.117
Active	23.66	24.08	0.81	19.54^b^	28.20^a^	<0.01	0.81	1.307

IR, infrared beak treated.

C, sham untreated control.

Comfort = preening + wing flapping + stretching.

Exploratory = foraging + environment pecking.

Low incidence = flip + head shaking + beak rubbing + strutting + unknown.

Active = standing + walking.

a,b Means within a main effect with different superscripts are significantly different (*P* ≤ 0.05).

Gender had a much larger impact on behavior over the 12-wk period compared to beak treatment (Table 6). Females spent more time resting than males at 1-d of age (60.08 vs. 56.55 percent of time, respectively), performing comfort behaviors at 8 d of age (1.32 vs. 0.95 percent of time, respectively), at the feeder at 8 wk of age (3.42 vs. 2.20 percent of time, respectively), and performing active and low incidence behaviors at 8 and 12 wk of age. Females spent less time resting compared to males at 8 and 12 wk of age.

At 6 d of age, infrared beak treatment affected how each gender responded with respect to the percent of time spent performing comfort behaviors (preening, wing flapping, and wing stretching). Female C turkeys spent more time (1.53%) performing these behaviors compared to all other treatments (1.15, 1.22, 1.12 percent of time, for female IR, male IR, and male C turkeys, respectively).

### Histology

**Figure 1:**
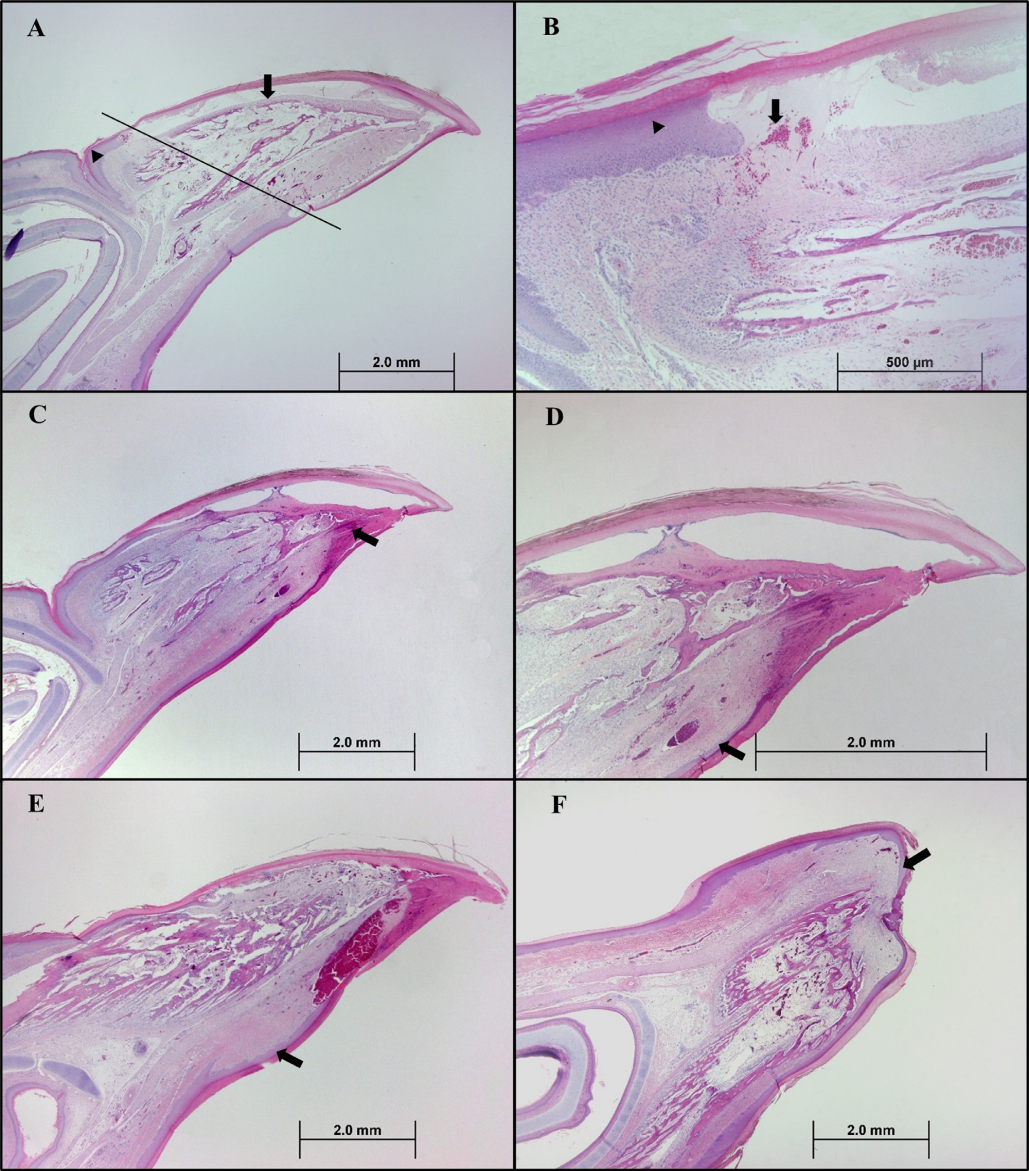
(A) Female turkey d-1 post-infrared (IR) beak treatment at 2× magnification. Coagulative necrosis of the beak epithelium and internal tissue anterior to the line where the IR beam penetrates through the beak. Arrowhead indicates normal beak epithelium. Arrow indicates necrotic beak epithelium. (B) Male turkey d-1 post-IR beak treatment at 10× magnification. Coagulative necrosis of the beak epithelium. Hemorrhage (arrow). Normal epithelium (arrowhead). (C) Male turkey d-5 post-IR beak treatment at 2× magnification. Formation of a serum clot between normal and necrotic beak tissue (arrow). (D) Male turkey d-5 post-IR beak treatment at 4× magnification. Formation of new beak epithelium (arrow). (E) Male turkey d-9 post-IR beak treatment at 2× magnification. Progression of formation of new beak epithelium (arrow). (F) Female turkey d-15 post-IR beak treatment at 2× magnification. New beak epithelium is about to unite at the beak tip (arrow).

At 1-d post-beak treatment, beaks showed coagulative necrosis of the epithelium and tissue below the treatment line. Hemorrhage and edema were also observed (Figures 1A and 1B). By 5 d post-treatment, the formation of new beak epithelium was visible (Figures 1C and 1D). Formation of new beak epithelium continued to progress at 9 d of age although the beak tip had not yet sloughed, and necrotic debris remained at the beak tip (Figure 1E). At 15 d of age, the beaks of both male and female IR turkeys had sloughed. Minimal necrotic debris was observed at the beak tip and the new beak epithelium was about to unite at the beak tip (Figure 1F). Both male and female IR turkeys showed complete healing of the beak tissue by 20 d of age. None of the IR beaks sampled showed evidence of post-treatment neuroma formation or abnormal nerve growth. Bacteria were observed in the necrotic tissue at the beak tip; however, there was no bacteria observed within the healed beak tissue.

## DISCUSSION

There is significant pressure to eliminate the practice of beak treatment in all commercial poultry species, with many European countries banning any form of treatment while others allow only infrared beak treatment until reliable alternatives are available (Council Directive, 1999; Department for Environment Food and Rural Affairs, 2010; Scottish Executive, 2010). Infrared beak treatment is reported to be more welfare-friendly compared to older methods (Gentle and McKeegan, 2007; Dennis et al., 2009; Struthers et al., 2019b); however, most previous research has been conducted using laying hens and it is still not fully understood how turkeys respond to infrared beak treatment.

One of the concerns regarding any form of beak treatment is that it may cause stress and pain to the bird (Kuenzel, 2007). Alterations in the number of circulating heterophils and lymphocytes have been associated with decreased immune function and can also be an indicator of stress (Gross and Siegel, 1983; Maxwell, 1993). In the present study, there was no evidence of stress in the IR treatment based on the H/L ratio when compared to C turkeys. At 20 d of age, turkeys with intact beaks had higher H/L ratios compared to IR turkeys. This difference may be due to increased aggression in the untreated turkeys resulting in a stress response. Although there was no difference in the amount of time spent performing aggressive behaviors between treated and untreated turkeys, this does not necessarily mean these behaviors were not being performed. It is more likely that the scan sampling technique used in the current study has a limited ability to detect low frequency and short duration behaviors like aggressive pecking (Rose, 2000). An increase in aggressive behavior is further supported by the fact that the first instances of turkeys requiring treatment for injurious pecking occurred only a few days later at 26 d of age (Struthers et al., in review). This suggests that infrared beak treatment reduced the damage that birds could inflict upon each other, and therefore, reduced the amount of stress the birds were experiencing. In relation to the second concern associated with beak treatment, the pecking force data from the present study suggests that the treated turkeys were not in pain post-beak treatment. This is further supported by the behavioral data, in which no differences were found for the percent of time birds spent performing beak-related behaviors such as feeding, drinking, preening, and exploratory pecking. This is like what has been reported in infrared beak-treated laying hens (Gentle and McKeegan, 2007; Freire et al., 2008; Struthers et al., 2019b).

Previous research has shown that infrared beak treated hens consistently have better feather cover compared to their untreated counterparts (Morrissey et al., 2016; Riber and Hinrichsen, 2017; Struthers et al., 2019b). This can have important consequences for both bird welfare and performance. The loss of feathers and areas of bare skin increases the risk of wounds and cannibalism and can cause birds to direct more energy toward thermoregulation and less toward growth, resulting in increased feed costs and poor feed efficiency (Leeson and Walsh, 2004). In the present study, infrared beak treatment did not have a consistent effect on feather cover over the 12-wk period. At 8 wk of age, C turkeys had better feather cover overall, but only in males, while at 12 wk, infrared beak treatment improved overall feather cover, but only in females.

Beak treatment had minimal and inconsistent effects on behavior during the first 3 wk of rearing. Previous studies have reported that birds with intact beaks spend more time exploring their environment compared to those that were beak treated (either by hot-blade trimming or infrared beak treatment) (Gentle and McKeegan, 2007; Struthers et al., 2019b) and this was observed in the present study with C turkeys spending more time exploratory pecking compared to IR turkeys at 6 d of age. It is thought that exploratory behavior is expressed when birds are not in pain and their basic needs are being met (Duncan, 1998). However, both Gentle and McKeegan (2007) and Struthers et al., (2019b) found no behavioral evidence of pain, which suggests that the increased exploratory behavior in control birds resulted from other unknown factors. When taken in conjunction with the pecking force data, it appears unlikely that the IR turkeys were performing less exploratory pecking because of pain.

In the present study, IR turkeys were observed spending a greater percent of time resting compared to C turkeys 1 d post-beak treatment. Inactivity (i.e., an increase in resting and a decrease in standing/walking) following beak treatment may be an indicator of pain or discomfort (Cunningham et al., 1992; Marchant-Forde et al., 2008). Marchant-Forde et al. (2008) found that pullets with intact beaks spent less time standing resting compared to infrared beak-treated pullets but only for a period of up to 4 d post-treatment. Although there were differences in time spent resting in the present study, there was not a corresponding decrease in time spent standing or walking in the IR turkeys. There were no other indications that the IR turkeys were in pain at this age as there were no differences in beak-related behaviors or H/L ratios. The difference in resting behavior also disappeared by 6 d of age. The histology of the treated turkey beaks in the present study followed a similar pattern to that observed in both hot-blade trimmed (Gentle et al., 1997) and infrared beak-treated laying hens (McKeegan and Philbey, 2012; Struthers et al., 2019a). In the present study, no abnormal neuroma formations were noted.

In conclusion, infrared beak treatment had a minimal impact on the welfare of male and female turkeys reared to 12 wk of age. Infrared beak treatment did not appear to cause acute or chronic pain in the turkeys as there was no evidence of neuroma formation or abnormal nerve growth. The results of the present study support the continued use of infrared beak treatment to help improve turkey welfare by reducing the damage that birds can inflict upon each other until reliable and effective alternatives to beak treatment are found.
